# Early Development of the Gonadotropin-Releasing Hormone Neuronal Network in Transgenic Zebrafish

**DOI:** 10.3389/fendo.2013.00107

**Published:** 2013-08-30

**Authors:** Yali Zhao, Meng-Chin A. Lin, Matthew Farajzadeh, Nancy L. Wayne

**Affiliations:** ^1^Department of Physiology, David Geffen School of Medicine, University of California Los Angeles, Los Angeles, CA, USA

**Keywords:** brain, development, embryo, gonadotropin-releasing hormone, kisspeptin, neuron, teleost

## Abstract

Understanding development of gonadotropin-releasing hormone (GnRH) neuronal circuits is fundamental to our understanding of reproduction, but not yet well understood. Most studies have been focused on GnRH neurons located in the hypothalamus and preoptic area (POA), which directly regulate the pituitary-gonadal axis. In zebrafish (*Danio rerio*), two forms of GnRH have been identified: GnRH2 and GnRH3. GnRH3 neurons in this species plays two roles: hypophysiotropic and neuromodulatory, depending on their location. GnRH3 neurons in the ventral telencephalon, POA, and hypothalamus control pituitary-gonadal function; in other areas (e.g., terminal nerve), they are neuromodulatory and without direct action on reproduction. To investigate the biology of GnRH neurons, a stable line of transgenic zebrafish was generated in which the GnRH3 promoter drives expression of a bright variant of green fluorescent protein (Emerald GFP, or EMD). This provides unprecedented sensitivity in detecting and imaging GnRH3 neurons during early embryogenesis in the transparent embryo. Using timelapse confocal imaging to monitor the time course of GnRH3:EMD expression in the live embryo, we describe the emergence and development of GnRH3 neurons in the olfactory region, hypothalamus, POA, and trigeminal ganglion. By 50 h post fertilization, these diverse groups of GnRH3 neurons project broadly in the central and peripheral nervous systems and make anatomical connections with each other. Immunohistochemistry of synaptic vesicle protein 2 (a marker of synaptic transmission) in this transgenic model suggests synaptic formation is occurring during early development of the GnRH3 neural network. Electrophysiology reveals early emergence of responsiveness to the stimulatory effects of kisspeptin in terminal nerve GnRH3 neurons. Overall, our findings reveal that the GnRH3 neuronal system is comprised of multiple populations of neurons as a complicated network.

## Introduction

Gonadotropin-releasing hormone (GnRH) neurons form the final common pathway for the central regulation of fertility. In vertebrate species, 15 variants of GnRH have been identified (1, 2). Each species studied has two or three forms of GnRH with distinct genes, locations, and functions (3). In most vertebrates, GnRH1 is mainly synthesized and released from a group of neurons located in the preoptic area (POA) and hypothalamus, and is essential for controlling pituitary-gonadal functions (4). GnRH2 located in midbrain tegmentum and GnRH3 in terminal nerve associated with the olfactory region are not hypophysiotropic; rather they play neuromodulatory roles that may be indirectly linked to the reproductive neuroendocrine axis (5, 6). The zebrafish genome is missing an ortholog to mammalian GnRH1, but expresses GnRH2 in midbrain tegmentum and GnRH3 widely in forebrain areas of terminal nerve, ventral telencephalon, POA, and hypothalamus (7, 8). Similar to GnRH1 neurons in other vertebrates, GnRH3 neurons located in ventral telencephalon, POA, and hypothalamus are thought to play hypophysiotropic roles in control of reproduction in zebrafish (7, 9). There is the curious and under-appreciated observation that GnRH3 is also expressed in the trigeminal ganglion of the peripheral nervous system of medaka and zebrafish embryos (10, 11). Presumably, this peripheral population of GnRH3 neurons, along with that in the terminal nerve associated with the olfactory region, play neuromodulatory functions that are not directly related to reproduction.

Earlier studies in mammals showed that GnRH neurons are born in the nasal/olfactory placode, and then migrate into the forebrain during embryogenesis to eventually settle in the POA-hypothalamus (12–15). This issue has come under renewed scrutiny as a result of studies in zebrafish suggesting multiple origins of GnRH neurons – that terminal nerve GnRH3 neurons originate from developing cranial neural crest, while the hypophysiotropic population is from the anterior pituitary placode (16). However, more recent work in zebrafish suggests that the POA-hypothalamic and terminal nerve populations of GnRH3 neurons both originate from the olfactory region (9). This controversy serves to emphasize the importance of understanding the origin and developmental processes of GnRH neurons, as defects in GnRH neuron birth and migration ultimately lead to reproductive abnormalities (17, 18).

Zebrafish has become an increasingly popular species to use for understanding mechanisms regulating embryonic development because the embryos are transparent, allowing visualization of organ and cellular development in the intact embryo in real time. Further, the zebrafish genome has been cloned, providing information about the molecular biology of the organism. In the present work, we used a stable line of transgenic zebrafish generated by our laboratory, in which the GnRH3 promoter drives expression of a bright variant of green fluorescent protein (Emerald GFP, or EMD). This provides unprecedented sensitivity in detecting and imaging GnRH3 neurons in the live, intact embryo (19). In the present study, we continue investigations of the morphological development of the complex GnRH3 neural network using confocal microscopic imaging of live GnRH3:EMD transgenic zebrafish embryos from the time of first expression of GnRH3 through early embryonic development. We also began testing the hypotheses that: (1) Multiple populations of GnRH3 neurons develop connectivity with each other; (2) Kisspeptin regulates spontaneous action potential firing of GnRH3 neurons during embryogenesis; and (3) GnRH3 neurons exhibit the potential for synaptic communication during early embryonic development.

## Materials and Methods

Unless otherwise stated, all chemicals were purchased from Sigma-Aldrich, Inc. (St. Louis, MO, USA).

### Animals

Wild type and GnRH3:EMD transgenic lines of brass zebrafish were used in this study. Generation of gene constructs and transgenic fish have been described previously (19). Animals were maintained in a zebrafish aquarium system on a 14L:10D photoperiod at 28°C. Fish were fed twice daily with flake food and live brine shrimp.

Timed breeding was organized in order to collect embryos with a known fertilization time. The day before breeding, a pair of adult GnRH3:EMD male and female fish were divided in a tank with a physical barrier. After lights on the next morning, the divider was removed allowing the fish to mate – which happens very quickly. Fertilized eggs were immediately collected and maintained in a Petri dish filled with embryonic water at 28.5°C. The timing of hours post fertilization (hpf) was the time that the dividers were removed for mating. All procedures were carried out in accordance with and approved by the Animal Care and Use Committee of UCLA.

### Confocal imaging

Live embryos were dechorionated manually, embedded in a drop of 0.8% agarose (Fisher Scientific, Pittsburgh, PA, USA) in glass-bottom culture dishes (35 mm diameter with 14 mm glass, No. 1.5 thickness; MatTek Corp., Ashland, MA, USA) and oriented using blunt forceps (lateral-side or ventral-side up, depending on which populations of GnRH3 neurons were being imaged). Once the agarose solidified, fish saline was added to keep the agarose moist. Embryos were then viewed under an upright Olympus confocal microscope and imaged using the Fluoview imaging system (Olympus America Inc., Center Valley, PA, USA). Images were taken using a water immersion 20× or 40× objective. EMD fluorescence was observed using an Argon laser (488 nm) with an emission barrier filter of 510 nm. Optical sections were made every 0.5 μm along the *z*-axis from the dorsal to the ventral side of the embryo. Images were achieved through projections of the *z*-stack. Timelapse images were acquired by scanning the embryos every 15 or 30 min. To follow the development of a specific group of GnRH3 neurons, we used a 40× objective with digital zoom (additional 2× or 3×) and took *z*-stack images every 15 min. The projection images were compiled in chronological order to form timelapse movies using Windows Movie Maker (Microsoft Inc., Redmond, WA, USA). *Z*-stack images were processed for projection, three-dimensional animations, movies, and analysis. To control the quality of images, parameters of the Fluoview program and microscope were adapted, but kept constant for the same set of experiments.

In some experiments (shown in Figures 3B and 4B), embryos were anesthetized with ethyl 3-aminobenzoate methanesulfonate salt (MS-222) and fixed at different stages with 4% paraformaldehyde as describe below, washed with PBS, and embedded in 0.8% agarose in lateral or ventral positions so as to image in the same way as the live embryos. Imaging fixed embryos provided a “snap-shot” view of different populations of GnRH3 neurons at known critical developmental times based on the live imaging, and allowed for more accurate analysis of changes in neuron number.

GnRH:EMD neurons were counted through the use of ImageJ processing software (ImageJ, free software from National Institutes of Health). Using ImageJ, contrast and brightness of the confocal *z*-stack images were adjusted in order to best visualize the individual cell bodies in a given neuronal population. Because it was difficult to determine the total number of cells in a 2-D projection image, each 0.5 μm optical slice in the *z*-stack was also individually analyzed. After the number of observed cell bodies was counted in each optical slice, the 3-D animation feature was used to confirm the number of neurons.

### Immunohistochemistry

SV2 immunohistochemistry is widely used in zebrafish as a presynaptic marker for monitoring synaptogenesis at early stages of development (20–22). Embryos at 25 and 50 hpf were fixed in 4% paraformaldehyde at 4°C overnight. Fixed embryos were washed with PBS, permeabilized in ice cold acetone for 8 min, rinsed in 0.3% Tween solution in PBS, then blocked for 2 h at room temperature in the dark with a solution of 0.3% Tween and 10% goat serum (in PBS). Following blocking, sections were washed and incubated overnight at 4°C with the primary antibody (anti-SV2, synaptic vesicle protein 2, mouse, 1:200) diluted in the blocking solution (23). Anti-SV2 antibody was purchased from Developmental Studies Hybridoma Bank, University of Iowa (Iowa City, IA, USA). After washing with 0.3% Tween in PBS, embryos were incubated in secondary antibody (Alexa Fluor 594 anti-mouse, 1:1000; Invitrogen, Carlsbad, CA, USA) at room temperature for 1 h, followed by rinsing with PBS five times (10 min for each rinse). Fixed embryos were mounted in 0.8% agarose at lateral or ventral positions, covered with PBS, then imaged with the confocal imaging system described above. Images were taken using a 40× water objective. EMD fluorescence was observed using an Argon laser (488 nm) with an emission barrier filter of 510 nm. Alexa Fluor 594 (secondary antibody staining) was visualized using a HeNe laser (543 nm) with an emission filter of 560–600 nm. All images were captured using the sequence mode to eliminate bleed-through of fluorescent signals. Images were taken in the *z*-plane at 0.5 μm steps. Specificity of immunostaining was confirmed using control embryos that were processed the same as experimental animals, but without the primary antibody.

### Electrophysiology and kisspeptin treatment

Loose-patch extracellular electrophysiological recordings of terminal nerve GnRH3 neurons from 2 to 3 dpf GnRH3:EMD live embryos were carried out as previously described (19). Briefly, the embryos were dechorionated and anesthetized by immersion in MS-222 (150 mg/l) and *d*-Tubocurarine (10 μM). They were glued ventral-side up to a glass coverslip at the bottom of a flow-through recording chamber (P1; Warner Instrument Corp., Hamden, CT, USA). Fine-tip forceps are used to gently remove the skull and expose the brain. Terminal nerve GnRH3:EMD neurons were identified using a 40× objective on an upright microscope (BX50W, Olympus, Melville, NY, USA). EMD-expressing neurons were targeted for loose-patch electrophysiology and recorded with a low-resistance seal (30–100 MΩ) in current clamp mode. After 5 min of stable baseline recording, 100 nM kisspeptin1(10) was bath applied for 5 min and spontaneous action potential firing was continuously recorded. Kisspeptin1(10) is a biologically active 10-amino-acid sequence (synthesized by Bachem Inc., Torrance, CA, USA) previously described for treatment of fish neural tissue by Zhao and Wayne (24). Both loose-patch and whole-cell electrophysiology following the procedure described by Ramakrishnan et al. (19) was used to record membrane potential (Voltmeters) and spontaneous action potential firing from trigeminal GnRH3:EMD neurons from live, intact embryos (25–30 hpf). Embryos were glued in the recording chamber in a lateral position so as to best expose the GnRH3:EMD-expressing neurons in the trigeminal ganglion. Neuron electrical activities were monitored and recorded by using both AxoGraph software (Axon Instruments, Foster City, CA, USA) and PowerLab data acquisition and analysis instrumentation and software (ADInstruments Inc., Colorado Springs, CO, USA). Subsequent data analysis of spike frequency was performed using AxoGraph software. Temperature in the recording chamber was maintained at 21–22°C throughout the experiments.

### Statistical analysis

Values are shown as the mean ± SEM. Data were analyzed using Prism (version 5.0; GraphPad Software, Inc., San Diego, CA, USA). Differences were considered significant if *p* < 0.05. Experiments in which there were only two groups, statistical analysis was performed using Student’s *t*-test. Experiments in which there were multiple groups, statistical analysis was performed using ANOVA followed by *post hoc* Student’s *t*-test.

## Results

### Emergence of multiple populations of GnRH3 neurons in the central and peripheral nervous systems

Previous work using GnRH3:eGFP and GnRH3:EMD transgenic zebrafish showed multiple populations of neurons expressing green fluorescence. Validating that these neurons are *bone fide* GnRH3 neurons depends on co-localization of green fluorescence with either GnRH3 peptide (e.g., immunohistochemistry) or mRNA (e.g., *in situ* hybridization). Using these approaches, it was confirmed that neurons in the terminal nerve/olfactory region, hypothalamus, POA, ventral telencephalon, and trigeminal ganglion express endogenous GnRH3 (11, 19).

**Figure 1:**
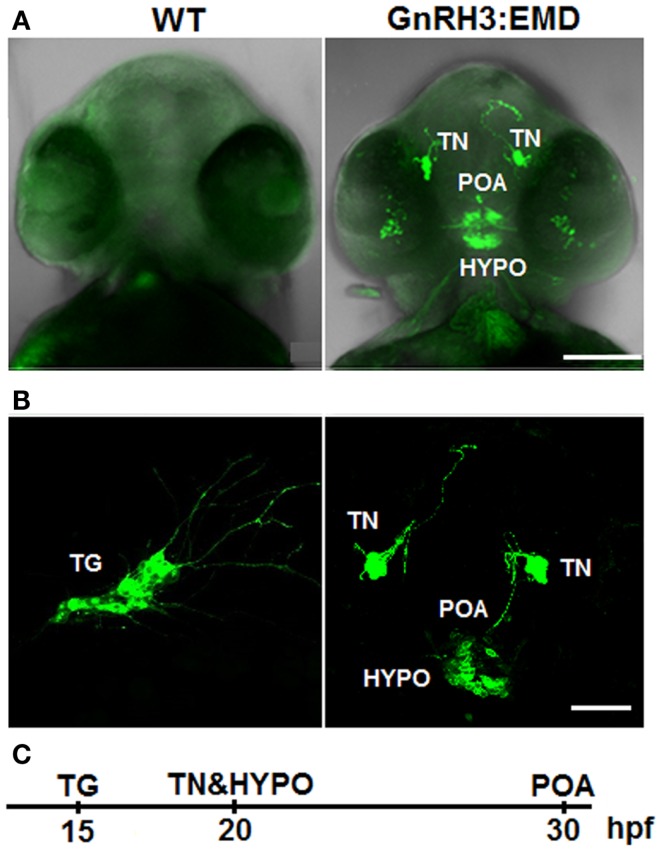
**Expression of GnRH3:EMD in live zebrafish embryos**. **(A)** *Z*-stack projection of confocal images of wildtype (WT) and GnRH3:EMD embryos at 40 hpf (ventral view). GFP and brightfield images were merged so that the head is visible. **(B)** GFP confocal images of multiple populations of GnRH3:EMD-expressing neurons. Left: lateral view of GnRH3:EMD neurons expressed in the trigeminal ganglion (TG) at 20 hpf. Right: ventral view of GnRH3:EMD neurons expressed in terminal nerve (TN), preoptic area (POA), and hypothalamus (HYPO) at 30 hpf stage. **(C)** Timeline of the GnRH3:EMD neurons’ emergence. Scale bar: **(A)** 100 μm; **(B)** 50 μm.

Figure 1A shows live images of a ventral view of the whole head of two zebrafish embryos at 40 hpf. Both images are shown with the merging of brightlight and GFP fluorescence stimulation so that the positioning of GnRH3:EMD-expressing neurons can be visualized relative to the eyes and nose. The image on the right shows populations of GnRH3:EMD-expressing neurons in the central nervous system of a transgenic zebrafish: terminal nerve, POA, and hypothalamus. The image on the left shows a wildtype embryo, exposed to the same UV light as the transgenic embryo – with little to no green autofluorescence at this age. Although there was GnRH3:EMD expression emerging in retina at around 30 hpf, we have so far not been able to verify that these neurons contain endogenous GnRH3. Failure to show endogenous GnRH3 mRNA may be due to these neurons showing such low expression of message that it is undetectable using *in situ* hybridization, or that the transgene is being ectopically expressed in these retina neurons. Nevertheless, it is notable that the retina is a target of GnRH3 neurons (11, 25, 26), expresses GnRH receptors (27, 28), and its development is disrupted when *GnRH3* gene expression is knocked down (11, 29).

Figure 1B shows live transgenic zebrafish embryos imaged in different positions so that different populations of GnRH3:EMD neurons can be more easily viewed. The panel on the left shows a lateral view at 20 hpf, with emergence of a group of GnRH3:EMD neurons in the trigeminal ganglion of the peripheral nervous system [first described and validated by (11)]. The panel on the right shows a ventral view of the emergence of the terminal nerve, POA, and hypothalamic populations of GnRH3:EMD neurons at 30 hpf. Figure 1C shows a timeline of the first emergence of different populations of GnRH3:EMD neurons, based on timelapse analysis of development of five transgenic embryos. The first population to emerge were those in the trigeminal ganglion at 15 hpf, then the terminal nerve and hypothalamic populations emerged about 5 h later at 20 hpf, followed by the POA population at about 30 hpf. This is an earlier time course than previously described by Abraham et al. (11). This can be explained by differences in the intensity of fluorescence between eGFP used by the Zohar group in their studies (11, 30, 31) and EMD used by the Wayne laboratory (19). Because EMD provides a stronger fluorescent signal than eGFP (32), it is a more sensitive marker of early GnRH neuron development.

### Early development of GnRH3 neurons in the trigeminal ganglion

**Figure 2:**
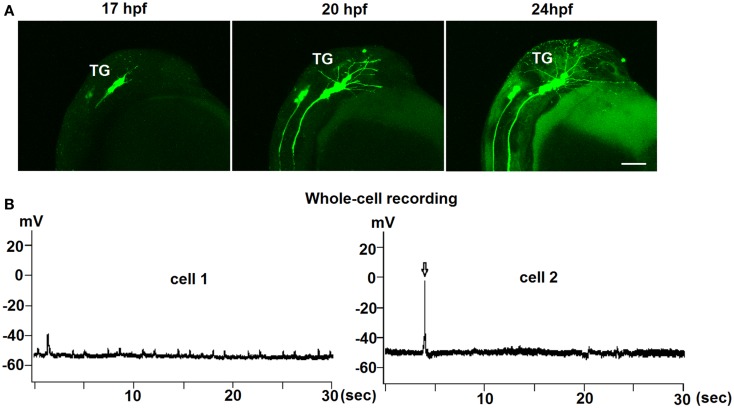
**Early development of trigeminal GnRH3:EMD neurons in live embryos**. **(A)** Representative lateral view of the trigeminal group of GnRH3:EMD neurons from a live embryo at 17, 20, and 24 hpf. Scale bar: 100 μm. **(B)** Sample traces of whole-cell electrophysiological recording from trigeminal GnRH3 neurons at 24 hpf (cell 1) and 27 hpf (cell 2). Arrow in the bottom panel shows an action potential.

Figure 2A shows a lateral view of the head of a live transgenic embryo from 17 to 25 hpf. GnRH3 neurons in the bilateral trigeminal ganglion are the first population to emerge, and very quickly send projections posterior into the spinal cord and anterior along the surface of the head. Movie S1 in Supplementary Material shows a timelapse movie with images taken every 30 min from 17 to 24 hpf. The movement of the projections is linear, with no back-and-forth meandering. Although there was GnRH3:EMD expression emerging in the spinal cord at around the same time as that of the trigeminal ganglion (data not shown), as with the retina, we have so far not been able to verify that these spinal cord neurons contain endogenous GnRH3.

To investigate if there was spontaneous electrical activity during early development, both loose-patch and whole-cell electrophysiological recordings were performed on trigeminal GnRH3:EMD neurons from 24 to 30 hpf embryos. Recordings were performed on seven neurons from six embryos (three whole cell and four loose-patch recordings) for approximately 10 min. Three neurons were electrically silent without any action potential firing during the recording period, one neuron showed two bursts (two to three spikes) of action potentials, and three neurons showed two to five spikes during the entire recording period. An earlier study also showed that trigeminal neurons in general show little to no spontaneous action potential firing in zebrafish embryos (33). Figure 2B shows electrophysiological recordings from two representative GnRH3 neurons in the trigeminal ganglion in 24 hpf (cell 1) and 27 hpf (cell 2) embryos. Whole-cell recordings revealed an average resting membrane potential (Vm) of −50 mV. In cell 2, there were three action potentials during a 9-min recording period. Although cell 1 did not fire action potentials, it did show depolarizing oscillations in Vm. This could be a reflection of an endogenous pacemaker potential as observed with terminal nerve GnRH3 neurons (34–36), or synaptically driven oscillations in Vm.

### Early development of GnRH3 neurons in the terminal nerve

**Figure 3:**
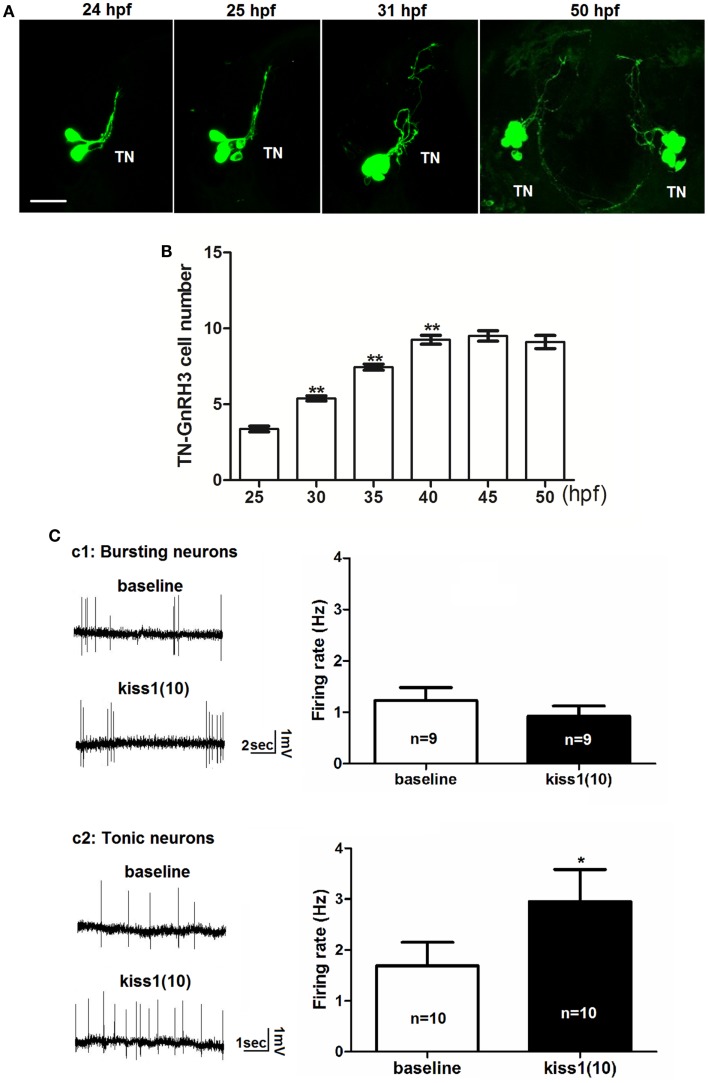
**Early development of terminal nerve (TN) GnRH3:EMD neurons**. **(A)** Representative confocal images (ventral view) of the left terminal nerve cluster at 24, 25, 30 hpf, and the bilateral clusters at 50 hpf. Scale bar: 20 μm. **(B)** Developmental change of neuron number per TN-GnRH3 cluster from 25 to 50 hpf. Embryos used for this study: 25 hpf, *n* = 27; 30 hpf, *n* = 39; 35 hpf, *n* = 18, 40 hpf, *n* = 20; 45 hpf, *n* = 5; 50 hpf, *n* = 10. **(C)** Loose-patch electrophysiological recording of spontaneous action potential firing of TN-GnRH3 (2–3 dpf) neurons. Neurons were recorded under baseline control conditions followed by treatment with a 10-amino-acid fragment of kisspeptin1. **(C1)** Immature bursting neuron; **(C2)**: mature tonically firing neuron. *p < 0.05 (3C2); **p < 0.001 compared to the previous time point **(B)**.

Figure 3A shows images of the development of one cluster of terminal nerve GnRH3 neurons between 24 and 31 hpf. During this time, there is an increase in the number of neurons expressing EMD, and neural projections are becoming more extensive. By 50 hpf (shown in the right panel with a view of the bilateral clusters), the number of GnRH3 neurons has stabilized, and the posterior projections between the bilateral clusters are in close apposition to each other (also shown in Figure 5A). Movie S2 in Supplementary Material shows a timelapse movie with images taken every 15 min from 24 to 31 hpf of a single cluster of terminal nerve GnRH3 neurons. During this time, anterior neural projections are meandering back-and-forth – very different from the much more linear movements of the projections from the trigeminal GnRH3 neurons shown in Movie S1 in Supplementary Material. Figure 3B shows the numbers of terminal nerve GnRH3 neurons between 25 and 50 hpf. There is a linear increase in cell number, which plateaus by 40 hpf. This is very similar to the time course of terminal nerve GnRH3 neuronal proliferation described by Gopinath et al. (37) using immunohistochemistry and *in situ* hybridization. We have done timelapse imaging of the terminal nerve GnRH3 neurons from a total of five embryos between 14 and 50 hpf, and have never seen any evidence of migration of neurons from this population to the hypophysiotropic populations as suggested by Abraham et al. (9).

We tested the hypothesis that kisspeptin, a neuropeptide that stimulates puberty and fertility through its actions on GnRH neurons (38–42), is also acting to stimulate the electrical activity of developing GnRH3 neurons during embryogenesis. Figure 3C shows the electrophysiological response of developing GnRH3 neurons in the terminal nerve to 100 nM kisspeptin1. Our earlier work showed that between 2 and 3 dpf, there is a maturation process by which zebrafish terminal nerve GnRH3 neurons become increasingly active (increase in number of neurons showing spontaneous action potential firing), and segue from an immature form of electrical activity (bursting pattern of firing) to a mature form of activity (tonic pattern of firing) (19). Figure 3C1 shows that there is no effect of kisspeptin1 on firing frequency of the GnRH3 neurons exhibiting the immature bursting form of action potential firing (baseline: 1.2 ± 0.2 Hz; kisspeptin: 0.9 ± 0.2; *n* = 9 neurons). However, the neurons exhibiting the mature tonic form of firing were stimulated by kisspeptin, showing a significant increase in firing frequency (baseline: 1.7 ± 0.5 Hz; kisspeptin: 3.0 ± 0.6 Hz, *p* < 0.05; *n* = 10 neurons) (Figure 3C2). This is very similar to the increase in firing rate of adult medaka terminal nerve GnRH3 neurons in response to treatment with 100 nM kisspeptin 1 (24).

### Early development of GnRH3 neurons in the POA and hypothalamus

**Figure 4:**
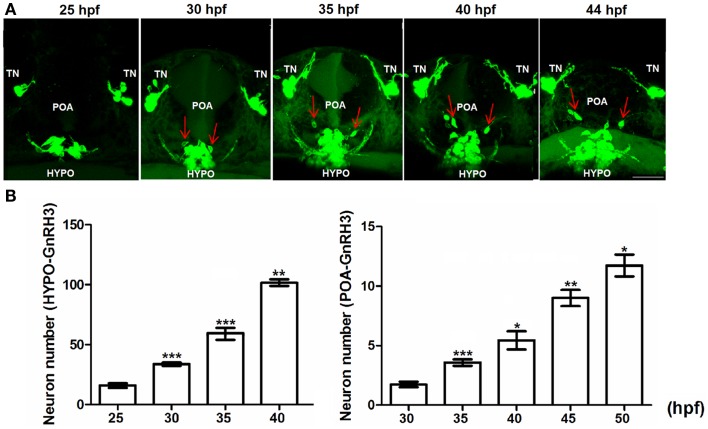
**Embryonic development of hypophysiotropic populations of GnRH3 neurons**. **(A)** Representative confocal images (ventral view) of the POA and hypothalamus (HYPO). Red arrows show GnRH3:EMD neurons migrating from hypothalamus toward the POA between 25 and 44 hpf. Scale bar: 50 μm. **(B)** Developmental change of GnRH3 neuron number in the POA (25–50 hpf) and HYPO (25–40 hpf). Embryos used for this study: 25 hpf, *n* = 27; 30 hpf, *n* = 39; 35 hpf, *n* = 18; 40 hpf *n* = 20; 45 hpf, *n* = 5; 50 hpf, *n* = 10. *p < 0.05, **p < 0.01, and ***p < 0.001 compared to the previous time points.

Figure 4 demonstrates the progressive development of the POA and hypothalamic populations of GnRH3 neurons between 25 hpf and 40–50 hpf. Figure 4A shows a ventral view of the head of a live embryo at different time points. Images of the POA and hypothalamic populations suggest that hypothalamic GnRH3 neurons emerge primarily *in situ*, and not from migrating neurons originating from the terminal nerve population as suggested by Abraham et al. (9). However, in 100% of timelapse images of this region, it was noted that some GnRH3 neurons migrated from the hypothalamic to the POA beginning at 28–29 hpf. This migration can be viewed in a timelapse movie shown in Movie S3 in Supplementary Material.

Figure 4B shows the rate of proliferation of GnRH3 neurons in the POA (right panel) and hypothalamus (left panel). By 40–50 hpf, the numbers have not yet reached a plateau – unlike what was shown with the terminal nerve neurons (Figure 4B). Also, the hypothalamic population has by far the greatest number of GnRH3 neurons during embryogenesis compared to the other populations.

### GnRH3 neuronal network and early synapse formation

**Figure 5:**
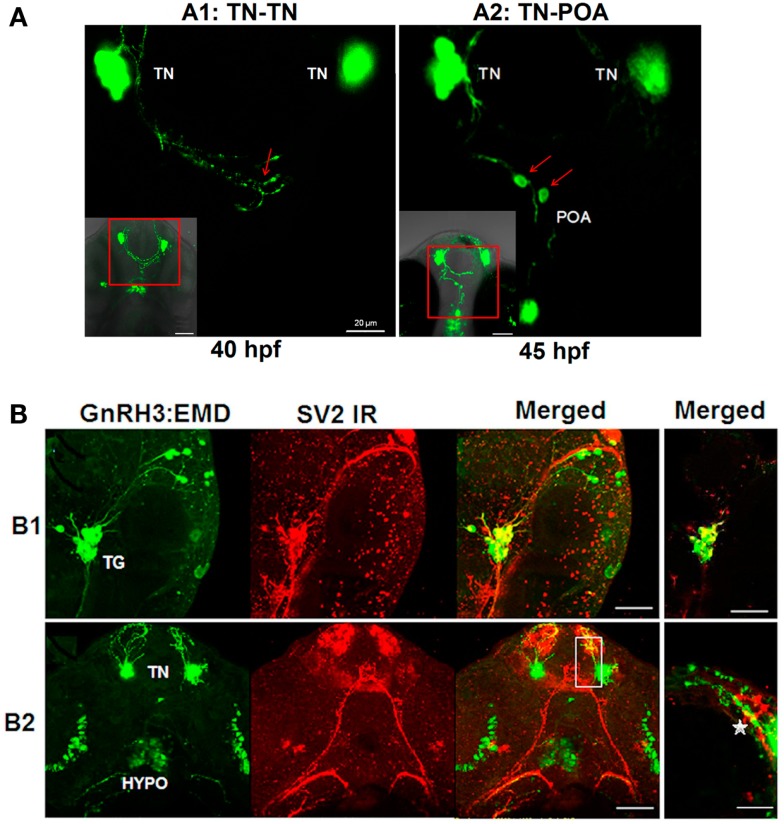
**Formation of GnRH3 neuronal circuits during embryogenesis**. **(A)** Representative confocal images (ventral view) showing close apposition of neural processes between GnRH3 populations. **(A1)** Example of connectivity between GnRH3 neuronal processes between two populations of the terminal nerve clusters (TN-TN). **(A2)** Example of connectivity between GnRH3 neuronal processes between terminal nerve and preoptic area populations (TN-POA). The insets in the left corners show *z*-stack projections of the confocal images; the larger panels show a single optical slice (0.5 mμm) from the inset image data. Arrows point to region of close apposition between neuron groups. Left: close apposition between neural processes from the bilateral TN-GnRH3 neuron clusters at 40 hpf. Right: close apposition between the neural process from a TN-GnRH3 neuron and the soma of a POA GnRH3 neuron at 45 hpf. Scale bar: insets, 50 μm; large panels, 20 μm. **(B)** Whole mount immunohistochemistry staining of synaptic vesicle protein 2 (SV2; red fluorescence) and GFP fluorescent imaging of GnRH3:EMD. **(B1)** Representative confocal image (lateral view) of trigeminal GnRH3:EMD neurons with SV2 staining at 25 hpf. Far left: trigeminal neurons expressing GnRH3:EMD; middle left: SV2 positive staining; middle right: merged image of GnRH3:EMD and SV2 fluorescence; far right: merged image from a single optical plane (0.5 μm). **(B2)** Representative confocal image (ventral view) of forebrain GnRH3:EMD neurons with SV2 staining at 50 hpf. Far left: GnRH3:EMD expression in terminal nerve (TN) and hypothalamus (HYPO); middle left: SV2 positive staining; middle right: merged image of GnRH3:EMD and SV2 fluorescence; far right: merged image from a single optical plane (0.5 μm) shown in the white box in the adjacent panel, and enlarged. The star shows an area of co-localization of GnRH3:EMD and SV2 in a neural process. In **(B1,B2)** yellow indicates regions of co-localization between GnRH3:EMD and SV2. Scale bar: 50 μm, except **(B1)** far right panel (20 μm).

Live imaging studies indicate that neural processes from one population of GnRH3 neurons form close appositions to processes and soma of GnRH3 neurons from other populations during embryogenesis (Figure 5). Ventral view of the developing head using confocal microscopy showed close apposition between: the neural processes of bilateral clusters of terminal nerve GnRH3 neurons at 40 hpf (Figure 5A1), and terminal nerve GnRH3 neural processes and POA GnRH3 soma at 45 hpf (Figure 5A2; Movie S4 in Supplementary Material showing three-dimensional rotating image).

SV2 is a membrane glycoprotein present in all synaptic vesicles and regulated secretory vesicles of endocrine cells, and is a marker of synaptic transmission (43–46). In the present study, immunohistochemical analysis of SV2 shows the expression of SV2 on GnRH3 neurons, suggesting that they are capable of synaptic communication during early embryogenesis (Figure 5B). Figure 5B1 shows a lateral view of the head to visualize GnRH3 neurons in the trigeminal ganglion at 25 hpf, and demonstrates co-localization of GnRH3:EMD and SV2 in the trigeminal neurons. Figure 5B2 shows a ventral view to visualize GnRH3 neurons in the forebrain at 50 hpf, and demonstrates co-localization of GnRH3:EMD and SV2 in neural processes originating from the terminal nerve population. There were less SV2 immunoreactive contacts on somas of terminal nerve GnRH3 neurons than on trigeminal GnRH3 neurons (SV2-ir positive somas: 25% of terminal nerve GnRH3 neurons; 100% of trigeminal GnRH3 neurons).

## Discussion

In this study, we use a bright variant of GPF (Emerald, or EMD) as a reporter of GnRH3 promoter activity to study the development of the GnRH3 neuronal network in transparent zebrafish embryos. Because of its unusual brightness, the EMD transgene provides a more sensitive method for detecting early expression of GnRH3 than previously reported studies using immunohistochemistry, *in situ* hybridization, and eGFP transgenes (19, 30, 37, 47, 48). Based on GnRH3:EMD expression in live transgenic zebrafish embryos, we have been able to map the earliest emergence and development of different populations of GnRH3 neurons in the central and peripheral nervous systems.

Basic understanding of the origin and developmental pathway of GnRH neural system in zebrafish – an important model system for understanding the molecular genetics of organ and cellular development – has become controversial. In the present study, careful analysis of the time course of GnRH3 neuron emergence and development reveals that there is no migration of neurons between terminal nerve and POA-hypothalamic populations. The exception was hypothalamic GnRH3 neurons migrating into the POA during embryogenesis. There wasn’t a single instance of a GnRH3 neuron migrating from the olfactory/terminal nerve region into the POA-hypothalamic region. Confocal imaging every 15–30 min from first detection of GnRH3 neurons in the olfactory region at 20 hpf until 50 hpf (well after the hypophysiotropic GnRH3 neurons have emerged) would have revealed neuron migration between these two regions, if it existed. This pattern of development more closely resembles that described by Whitlock et al. (47), in which terminal nerve GnRH3 neurons and hypophysiotropic GnRH3 neurons originate from independent locations – terminal nerve GnRH3 neurons from cranial neural crest, and hypophysiotropic GnRH3 neurons from adenohypophyseal placode. However, in an intriguing study by Abraham et al. (9), they showed that laser ablation of terminal nerve/olfactory GnRH3 neurons in embryonic zebrafish resulted in elimination of the hypophysiotropic populations of GnRH3 neurons (9). Although the authors interpreted their results to suggest that the olfactory region is the source of hypophysiotropic GnRH3 neurons, an alternative explanation is that key populations of GnRH3 neurons are required for survival of other GnRH3 neuron populations through transmission of electrochemical communication during development. Previous work showed that GnRH is neurotrophic for GnRH3 neurons and other neurons in zebrafish. Specifically, loss of function of GnRH3 using antisense morpholino knockdown interfered with normal brain and eye formation, as well as disrupted development of GnRH-neuron projections and localization of their somas in the zebrafish embryo (11, 29). This suggests that GnRH3 secretion plays an important role in the survival of select neuronal populations, including GnRH3 neurons. Our present findings support the hypothesis that trigeminal, terminal nerve, and hypophysiotropic populations of GnRH3 neurons originate independently, with no migration between terminal nerve and hypophysiotropic groups. However, their survival and development are co-dependent on transmission of neurochemical signals between the different populations of GnRH3 neurons. Interestingly, our *in vivo* imaging study suggests that POA groups may comprise two subpopulations, one originates *in situ*, and one migrates from the hypothalamus. This finding provides a supporting explanation for their functional similarities in control of reproduction.

Further support for the potential of neural transmission between populations of GnRH3 neurons comes from confocal imaging showing neural processes in close apposition between the bilateral terminal nerve clusters, and between the terminal nerve and POA GnRH3 cell bodies. We also show that developing GnRH3 neurons are capable of synaptic transmission as a function of their expression of SV2 required for exocytosis. Notably, the forebrain is heavily laced with SV2 expression, suggesting widespread synaptic transmission during embryogenesis. We observed that there were less SV2 immunoreactive contacts on the somas of GnRH3 neurons from the terminal nerve population than the trigeminal neuron population. This finding suggests differential development of synaptic transmission of these two groups of GnRH3 neurons, with relatively less synaptic input received by the terminal nerve population. This is the first study indicating the possibility of early synaptogenesis onto GnRH neurons in embryos, but co-localization of SV2 and GnRH using electron microscopy would be essential to confirm our findings. The present results showed SV2 in neural processes and cell bodies. Work using GnRH1 cell cultures from the nasal placode of rhesus monkeys showed that GnRH peptide was released from both cell bodies and their neural processes (49). In our model system, the presence of SV2 immunoreactivity on both soma and neural processes may be related to GnRH release, as well as regulation of GnRH neuron growth and development during embryogenesis.

An adult-like pattern of spontaneous action potential firing develops by 2–3 dpf in the terminal nerve GnRH3 neurons of zebrafish embryos (19). This early maturation process suggests that this population of neurons plays an important physiological role during embryogenesis, perhaps supporting the survival and development of other neurons through electrochemical communication. Work in mouse showed that treatment of embryonic POA explants with kisspeptin stimulated GnRH1 neurite growth (50), indicating that kisspeptin can play a role in development of GnRH neurons during embryogenesis in addition to its more well-known role in activating puberty and stimulating reproduction. We tested the hypothesis that kisspeptin is also playing a role in stimulating the electrical activity of GnRH neurons during embryogenesis. Two forms of the kiss gene (kiss1, kiss2) and the kiss receptor gene (kiss1r, kiss2r) have been identified and cloned from zebrafish, and their expression patterns described in adult brain (51–53). In total, the data suggest that as with mammals, the kisspeptin neural system in zebrafish plays an important role in regulating GnRH neurons. The unknown is if it plays a role in GnRH neuron development. The present findings indicate that the potential for kiss1 to stimulate electrical activity of terminal nerve GnRH3 neurons is dependent on the maturity of the cell during embryogenesis. Our earlier work showed that immature neurons showed a bursting pattern of action potential firing, whereas mature neurons showed a tonic beating pattern of firing (19). Wang and coworkers identified four different firing patterns in dissociated terminal nerve GnRH3 neurons in culture from adult transgenic zebrafish. These patterns were classified as rhythmic firing, burst firing, irregular firing, and rare firing (48). However, cultured neurons may show altered physiological characteristics compared to their functioning in the intact brain. Because adult zebrafish terminal nerve GnRH3 neurons are encased in a tough connective sheath, it has not been possible to perform electrophysiology on them in the intact nervous system. This is not the case with dwarf gourami and medaka, in which adult terminal nerve GnRH3 neurons are accessible for electrophysiological recording, and show a reliable tonic pattern of action potential firing in the intact brain (36, 54).

Our present results from 2 to 3 dpf zebrafish embryos shows that 100 nM kisspeptin1 significantly stimulated spike frequency from mature tonically firing GnRH3 neurons, but not from immature neurons showing a bursting pattern of action potential firing. Immature GnRH3 neurons may not respond electrically to kiss1 treatment if they lack sufficient synaptic inputs from interneurons expressing kisspeptin receptors. Importantly, studies in adult medaka show that the stimulatory actions of kisspeptin on GnRH3 neuron spike frequency are achieved indirectly through a synaptic transmission process (24). This is supported by work showing that kisspeptin receptors are expressed near GnRH neurons in the forebrain of adult medaka, but not actually on GnRH1–3 neurons (55). The observed stimulatory response in mature embryonic GnRH3 neurons is very similar to our recent findings in which 100 nM kisspeptin1 had a potent and long-lasting stimulatory effect on frequency of action potential firing of terminal nerve GnRH3 neurons from adult medaka fish (24). During embryogenesis, this stimulation of GnRH3 neuron spike frequency may serve to strengthen neural communication between populations of neurons, potentially enhancing neural development. Overall, our present findings coupled with earlier work suggest the emergence of a complex neural network of GnRH3 neurons that are interacting with other neurons during embryogenesis in such a way as to support mutual survival and optimal development.

## Conflict of Interest Statement

The authors declare that the research was conducted in the absence of any commercial or financial relationships that could be construed as a potential conflict of interest.

## Supplementary Material

The Supplementary Material for this article can be found online at http://www.frontiersin.org/Experimental_Endocrinology/10.3389/fendo.2013.00107/abstract

Supplementary Movie S1 **Timelapse movie showing the development of the trigeminal population of GnRH3:EMD neurons (lateral view) in a live zebrafish embryo (17–24 hpf)**.

Supplementary Movie S2 **Timelapse movie showing the development of the terminal nerve population of GnRH3:EMD neurons (ventral view) in a live zebrafish embryo (24–31 hpf)**.

Supplementary Movie S3 **Timelapse movie showing the development of GnRH3:EMD neurons in the live embryo and the neuron migration from hypothalamus to POA (25–44 hpf)**.

Supplementary Movie S4 **3-D animation of the image shown in Figure 5A2, illustrating the close apposition between neural processes of the terminal nerve GnRH3 neurons and the soma of a POA GnRH3 neuron**.

## References

[1] Lethimonier C Madigou T Muñoz-Cueto JA Lareyre JJ Kah O Evolutionary aspects of GnRHs, GnRH neuronal systems and GnRH receptors in teleost fish. Gen Comp Endocrinol (2004) 135:1–16 10.1016/j.ygcen.2003.10.007 14644639

[2] Kavanaugh SI Nozaki M Sower SA Origins of gonadotropin-releasing hormone (GnRH) in vertebrates: identification of a novel GnRH in a basal vertebrate, the sea lamprey. Endocrinology (2008) 149:3860–9 10.1210/en.2008-0184 18436713 PMC2488216

[3] Tostivint H Evolution of the gonadotropin-releasing hormone (GnRH) gene family in relation to vertebrate tetraploidizations. Gen Comp Endocrinol (2011) 170:575–81 10.1016/j.ygcen.2010.11.017 21118690

[4] Moenter SM Identified GnRH neuron electrophysiology: a decade of study. Brain Res (2010) 1364:10–24 10.1016/j.brainres.2010.09.066 20920482 PMC2992586

[5] Schneider JS Rissman EF Gonadotropin-releasing hormone II: a multi-purpose neuropeptide. Integr Comp Biol (2008) 48:588–95 10.1093/icb/icn018 21669818 PMC6283013

[6] Abe H Oka Y Mechanisms of neuromodulation by a nonhypophysiotropic GnRH system controlling motivation of reproductive behavior in the teleost brain. J Reprod Dev (2011) 57:665–74 10.1262/jrd.11-055E 22277963

[7] Steven C Lehnen N Kight K Ijiri S Klenke U Harris WA Molecular characterization of the GnRH system in zebrafish (*Danio rerio*): cloning of chicken GnRH-II, adult brain expression patterns and pituitary content of salmon GnRH and chicken GnRH-II. Gen Comp Endocrinol (2003) 133:27–37 10.1016/S0016-6480(03)00144-8 12899844

[8] Kuo MW Lou SW Postlethwait J Chung BC Chromosomal organization, evolutionary relationship, and expression of zebrafish GnRH family members. J Biomed Sci (2005) 12:629–39 10.1007/s11373-005-7457-z 16132106

[9] Abraham E Palevitch O Gothilf Y Zohar Y Targeted gonadotropin-releasing hormone-3 neuron ablation in zebrafish: effects on neurogenesis, neuronal migration, and reproduction. Endocrinology (2010) 151:332–40 10.1210/en.2009-0548 19861502

[10] Okubo K Sakai F Lau EL Yoshizaki G Takeuchi Y Naruse K Forebrain gonadotropin-releasing hormone neuronal development: insights from transgenic medaka and the relevance to X-linked Kallmann syndrome. Endocrinology (2006) 147:1076–84 10.1210/en.2005-0468 16293668

[11] Abraham E Palevitch O Ijiri S Du SJ Gothilf Y Zohar Y Early development of forebrain gonadotrophin-releasing hormone (GnRH) neurones and the role of GnRH as an autocrine migration factor. J Neuroendocrinol (2008) 20:394–405 10.1111/j.1365-2826.2008.01654.x 18208553

[12] Schwanzel-Fukuda M Pfaff DW Origin of luteinizing hormone-releasing hormone neurons. Nature (1989) 338:161–4 10.1038/338161a0 2645530

[13] Wray S Grant P Gainer H Evidence that cells expressing luteinizing hormone-releasing hormone mRNA in the mouse are derived from progenitor cells in the olfactory placode. Proc Natl Acad Sci U S A (1989) 86:8132–6 10.1073/pnas.86.20.8132 2682637 PMC298229

[14] Yamamoto N Uchiyama H Ohki-Hamazaki H Tanaka H Ito H Migration of GnRH-immunoreactive neurons from the olfactory placode to the brain: a study using avian embryonic chimeras. Brain Res Dev Brain Res (1996) 95:234–44 10.1016/0165-3806(96)00078-8 8874898

[15] Wierman ME Pawlowski JE Allen MP Xu M Linseman DA Nielsen-Preiss S Molecular mechanisms of gonadotropin-releasing hormone neuronal migration. Trends Endocrinol Metab (2004) 15:96–102 10.1016/j.tem.2004.02.003 15046737

[16] Whitlock KE Origin and development of GnRH neurons. Trends Endocrinol Metab (2005) 16:145–51 10.1016/j.tem.2005.03.005 15860410

[17] MacColl G Quinton R Bouloux PM GnRH neuronal development: insights into hypogonadotrophic hypogonadism. Trends Endocrinol Metab (2002) 131:12–8 11893524 10.1016/s1043-2760(01)00545-8

[18] Balasubramanian R Dwyer A Seminara SB Pitteloud N Kaiser UB Crowley WF Jr Human GnRH deficiency: a unique disease model to unravel the ontogeny of GnRH neurons. Neuroendocrinology (2010) 92:81–99 10.1159/000314193 20606386 PMC3214927

[19] Ramakrishnan S Lee W Navarre S Kozlowski DJ Wayne NL Acquisition of spontaneous electrical activity during embryonic development of gonadotropin-releasing hormone-3 neurons located in the terminal nerve of transgenic zebrafish (*Danio rerio*). Gen Comp Endocrinol (2010) 168:401–7 10.1016/j.ygcen.2010.05.009 20515692 PMC2922451

[20] Jonz MG Nurse CA Neuroepithelial cells and associated innervations of the zebrafish gill: a confocal immunofluorescence study. J Comp Neurol (2003) 16:1–17 10.1002/cne.10680 12722101

[21] Jontes JD Emond MR Smith SJ In vivo trafficking and targeting of N-cadherin to nascent presynaptic terminals. J Neurosci (2004) 13:9027–34 10.1523/JNEUROSCI.5399-04.2004 15483121 PMC6730076

[22] Ramesh T Lyon AN Pineda RH Wang C Janssen PM Canan BD A genetic model of amyotrophic lateral sclerosis in zebrafish displays phenotypic hallmarks of motoneuron disease. Dis Model Mech (2010) 3:652–62 10.1242/dmm.005538 20504969 PMC2931540

[23] Sakowski SA Lunn JS Busta AS Oh SS Zamora-Berridi G Palmer M Neuromuscular effects of G93A-SOD1 expression in zebrafish. Mol Neurodegener (2012) 7:44 10.1186/1750-1326-7-44 22938571 PMC3506515

[24] Zhao Y Wayne NL Effects of kisspeptin1 on electrical activity of an extrahypothalamic population of gonadotropin-releasing hormone neurons in medaka (*Oryzias latipes*). PLoS ONE (2012) 7:e37909 10.1371/journal.pone.0037909 22649563 PMC3359290

[25] Wirsig-Wiechmann CR Wiechmann AF Vole retina is a target for gonadotropin-releasing hormone. Brain Res (2002) 950:210–7 10.1016/S0006-8993(02)03039-1 12231246

[26] Servili A Herrera-Perez P Kah O Munoz-Cueto JA The retina is a target for GnRH-3 system in the European sea bass, *Dicentrarchus labrax*. Gen Comp Endocrinol (2012) 175:398–406 10.1016/j.ygcen.2011.11.007 22138555

[27] Robison RR White RB Illing N Troskie BE Morley M Millar RP Gonadotropin-releasing hormone receptor in the teleost *Haplochromis burtoni*: structure, location, and function. Endocrinology (2001) 142(5): 1737–43 11316736 10.1210/endo.142.5.8155 PMC2672947

[28] Grens KE Greenwood AK Fernald RD Two visual processing pathways are targeted by gonadotropin-releasing hormone in the retina. Brain Behav Evol (2005) 66:1–9 10.1159/000085043 15821344 PMC1167600

[29] Wu S Page L Sherwood NM A role for GnRH in early brain regionalization and eye development in zebrafish. Mol Cell Endocrinol (2006) 257-258:47–64 10.1016/j.mce.2006.06.010 16934393

[30] Palevitch O Kight K Abraham E Wray S Zohar Y Gothilf Y Ontogeny of the GnRH systems in zebrafish brain: in situ hybridization and promoter-reporter expression analyses in intact animals. Cell Tissue Res (2007) 327:313–22 10.1007/s00441-006-0279-0 17036230

[31] Abraham E Palevitch O Gothilf Y Zohar Y The zebrafish as a model system for forebrain GnRH neuronal development. Gen Comp Endocrinol (2009) 164:151–60 10.1016/j.ygcen.2009.01.012 19523393

[32] Han W Ng YK Axelrod D Levitan ES Neuropeptide release by efficient recruitment of diffusing cytoplasmic secretory vesicles. Proc Natl Acad Sci U S A (1999) 96:14577–82 10.1073/pnas.96.25.14577 10588747 PMC24478

[33] Douglass AD Kraves S Deisseroth K Schier AF Engert F Escape behavior elicited by single, channelrhodopsin-2-evoked spikes in zebrafish somoatosensory neurons. Curr Biol (2008) 18:1133–7 10.1016/j.cub.2008.06.077 18682213 PMC2891506

[34] Oka Y Tetrodotoxin-resistant persistent Na+ current underlying pacemaker potentials of fish gonadotrophin-releasing hormone neurones. J Physiol (1995) 482:1–6 7730975 10.1113/jphysiol.1995.sp020494 PMC1157748

[35] Oka Y Characterization of TTX-resistant persistent Na+ current underlying pacemaker potentials of fish gonadotropin-releasing hormone (GnRH) neurons. J Neurophysiol (1996) 75:2397–404 8793752 10.1152/jn.1996.75.6.2397

[36] Wayne NL Kuwahara K Aida K Nagahama Y Okubo K Whole-cell electrophysiology of gonadotropin releasing hormone (GnRH) neurons that express green fluorescent protein (GFP) in the terminal nerve of transgenic medaka (*Oryzias latipes*). Biol Reprod (2005) 73:1228–34 10.1095/biolreprod.105.042721 16107608

[37] Gopinath AL Tseng A Whitlock KE Temporal and spatial expression of gonadotropin releasing hormone (GnRH) in the brain of developing zebrafish (*Danio rerio*). Gene Expr Patterns (2004) 4:65–70 10.1016/S1567-133X(03)00149-2 14678830

[38] Seminara SB Messager S Chatzidaki EE Thresher RR Acierno JS Jr Shagoury JK The GPR54 gene as a regulator of puberty. N Engl J Med (2003) 349:1614–27 10.1056/NEJMoa035322 14573733

[39] Clarkson J d’Anglemont de Tassigny X Moreno AS Colledge WH Herbison AE Kisspeptin-GPR54 signaling is essential for preovulatory gonadotropin-releasing hormone neuron activation and the luteinizing hormone surge. J Neurosci (2008) 28:8691–7 10.1523/JNEUROSCI.1775-08.2008 18753370 PMC6670827

[40] Pielecka-Fortuna J Chu Z Moenter SM Kisspeptin acts directly and indirectly to increase gonadotropin-releasing hormone neuron activity and its effects are modulated by estradiol. Endocrinology (2008) 149:1979–86 10.1210/en.2007-1365 18162521 PMC2276721

[41] Oakley AE Clifton DK Steiner RA Kisspeptin signaling in the brain. Endocr Rev (2009) 30:713–43 10.1210/er.2009-0005 19770291 PMC2761114

[42] Oka Y Three types of gonadotrophin-releasing hormone neurones and steroid-sensitive sexually dimorphic kisspeptin neurones in teleosts. J Neuroendocrinol (2009) 21:334–8 10.1111/j.1365-2826.2009.01850.x 19210296

[43] Buckley KM Kelly RB Identification of a transmembrane glycoprotein specific for secretory vesicles of neural and endocrine cells. J Cell Biol (1985) 100:1284–94 10.1083/jcb.100.4.1284 2579958 PMC2113776

[44] Schivell AE Batchelor RH Bajjalieh SM Isoform-specific, calcium-regulated interaction of the synaptic vesicle proteins SV2 and synaptotagmin. J Biol Chem (1996) 271:27770–5 10.1074/jbc.271.44.27770 8910372

[45] Crowder KM Gunther JM Jones TA Hale BD Zhang HZ Peterson MR Abnormal neurotransmission in mice lacking synaptic vesicle protein 2A (SV2A). Proc Natl Acad Sci U S A (1999) 96:15268–73 10.1073/pnas.96.26.15268 10611374 PMC24809

[46] Boon KL Xiao S McWhorter ML Donn T Wolf-Saxon E Bohnsack MT Zebrafish survival motor neuron mutants exhibit presynaptic neuromuscular junction defects. Hum Mol Genet (2009) 18:3615–25 10.1093/hmg/ddp310 19592581 PMC2742401

[47] Whitlock KE Wolf CD Boyce ML Gonadotropin-releasing hormone (GnRH) cells arise from cranial neural crest and adenohypophyseal regions of the neural plate in the zebrafish, *Danio rerio*. Dev Biol (2003) 257:140–52 10.1016/S0012-1606(03)00039-3 12710963

[48] Wang X Huang L Li Y Li X Li P Ray J Characterization of GFP-tagged GnRH-containing terminalis neurons in transgenic zebrafish. J Cell Physiol (2011) 226:608–15 10.1002/jcp.22369 20717967

[49] Fuenzalida LC Keen KL Terasawa E Colocalization of FM1-43, Bassoon, and GnRH-1: GnRH-1 release from cell bodies and their neuroprocesses. Endocrinology (2011) 152:4310–21 10.1210/en.2011-1416 21896672 PMC3199012

[50] Fiorini Z Jasoni CL A novel developmental role for kisspeptin in the growth of gonadotrophin-releasing hormone neurites to the median eminence in the mouse. J Neuroendocrinol (2010) 22:1113–25 10.1111/j.1365-2826.2010.02059.x 20722977

[51] Biran J Ben-Dor S Levavi-Sivan B Molecular identification and functional characterization of the kisspeptin/kisspeptin receptor system in lower vertebrates. Biol Reprod (2008) 79:776–86 10.1095/biolreprod.107.066266 18509165

[52] Kitahashi T Ogawa S Parhar IS Cloning and expression of kiss2 in the zebrafish and medaka. Endocrinology (2009) 150:821–31 10.1210/en.2008-0940 18927220

[53] Servili A Le Page Y Leprince J Caraty A Escobar S Parhar IS Organization of two independent kisspeptin systems derived from evolutionary-ancient kiss genes in the brain of zebrafish. Endocrinology (2011) 152:1527–40 10.1210/en.2010-0948 21325050

[54] Oka Y Matsushima T Gonadotropin-releasing hormone (GnRH)- immunoreactive terminal nerve cells have intrinsic rhythmicity and project widely in the brain. J Neurosci (1993) 13:2161–76 7683049 10.1523/JNEUROSCI.13-05-02161.1993 PMC6576567

[55] Kanda S Akazome Y Mitani Y Okubo K Oka Y Neuroanatomical evidence that kisspeptin directly regulates isotocin and vasotocin neurons. PLoS ONE (2013) 8(4): e62776 10.1371/journal.pone.0062776 23638144 PMC3636218

